# Supplementary material for: Structural basis of GABARAP-mediated GABAA receptor trafficking and functions on GABAergic synaptic transmission
Source: Nat Commun. 2021 Jan 12;12:297. doi: 10.1038/s41467-020-20624-z (PMC7803741; doi:10.1038/s41467-020-20624-z)
Supplement: Supplementary file 1 — Supplementary Information [file 41467_2020_20624_MOESM1_ESM.pdf]

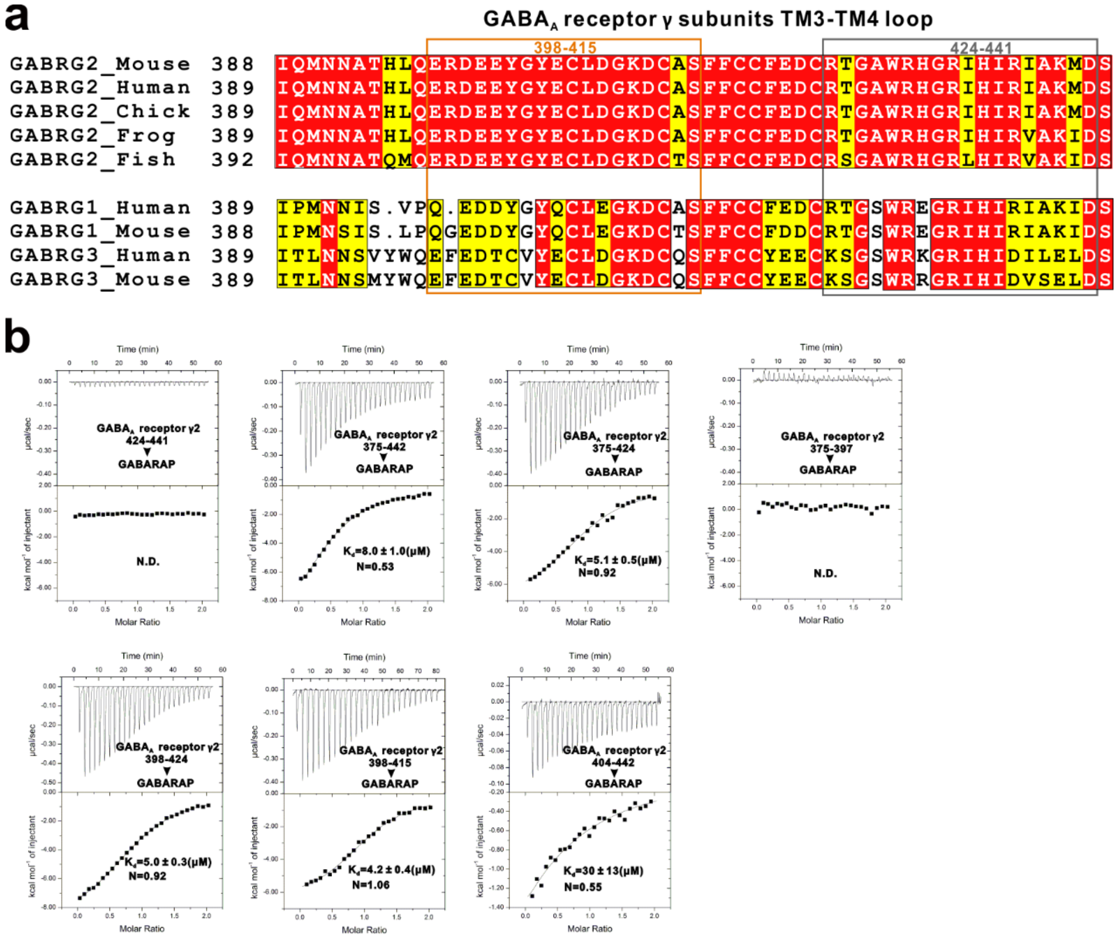

Supplementary Fig. 1 Biochemical mappings of the minimal GABARAP binding region in the γ2-GABA$_A$R.

(a) Amino acid sequence alignment of the γ2-ICD in several species and all the three γ subunits. Residues that are absolutely conserved or highly conserved are highlighted in red or yellow, respectively. **(b)** ITC-based mapping of the minimal GABARAP binding region in the γ2-GABA$_A$R. The minimal and complete GABARAP binding region identified is 398-415. The error of K_d for each ITC curve represents the curve fitting error. N: stoichiometry of binding. The 'N.D.' denotes that there is no detectable binding.

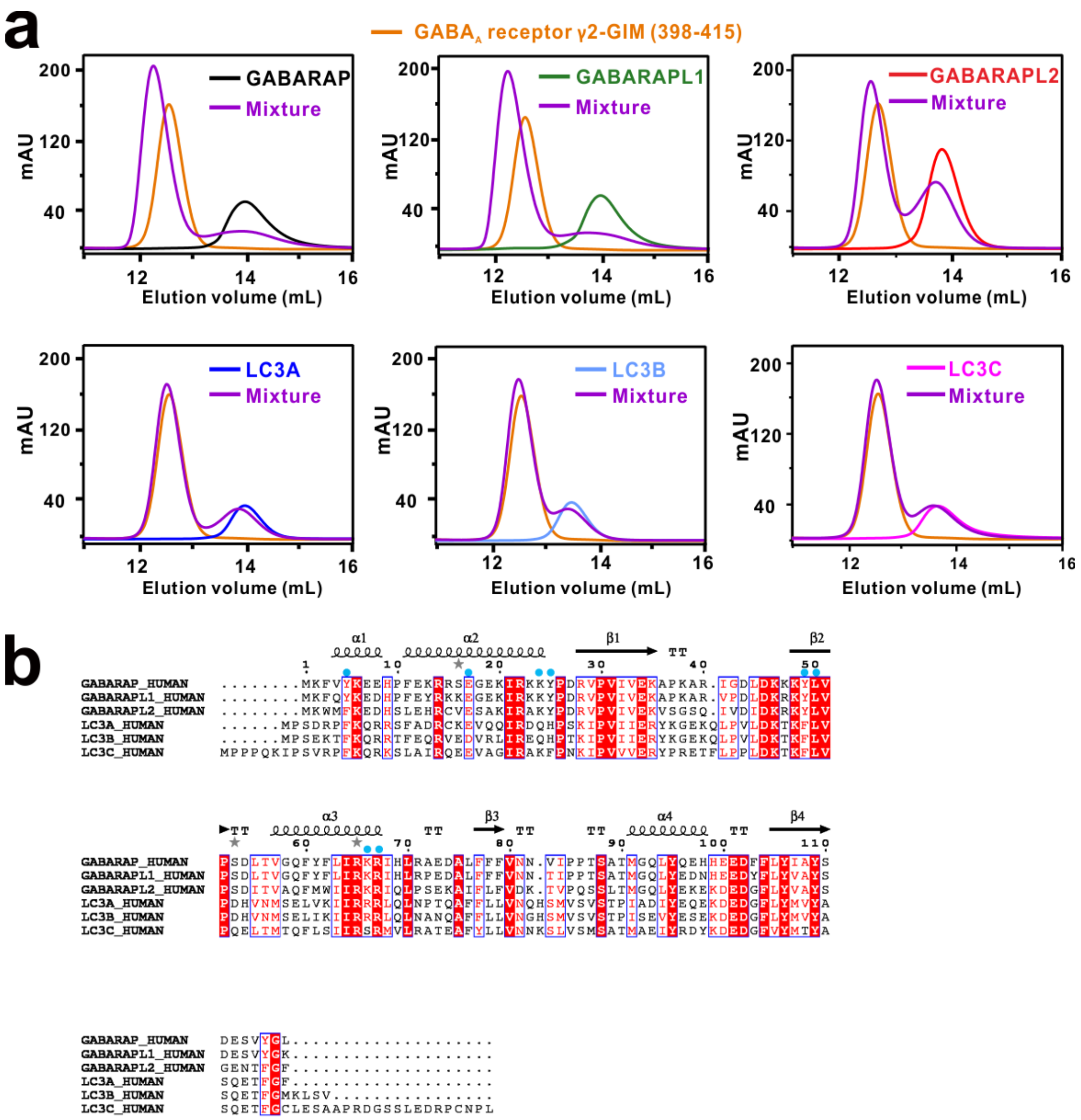

Supplementary Fig. 2 Biochemical characterizations of the interactions of GABA$_A$R γ2-GIM with different Atg8 members.

(a) Analytical gel filtration chromatography analysis of the interaction between purified GABA$_A$R γ2-GIM and 6 Atg8 family proteins. The mixture peak shifting towards the smaller elution volume indicates a complex formation between the two proteins mixed together. **(b)** Sequence alignment of six Atg8 members from human. The residues selected for mutagenesis or specificity analyses are indicated with cyan dots.

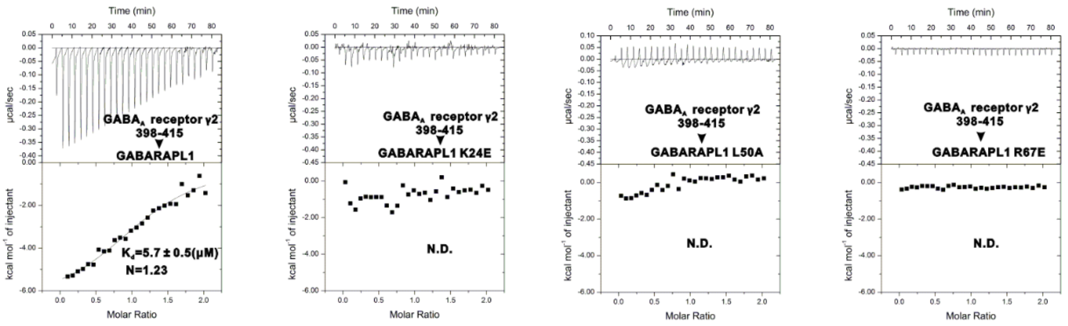

Supplementary Fig. 3 Validations of the GABARAPL1–γ2-GIM complex structure by mutations of key interface residues in GABARAPL1.

ITC results showing that mutating the key residues in GABARAPL1 decrease the binding to γ2-GIM when compared to the WT GABARAPL1. The error of K_d for each ITC curve represents the curve fitting error. N: stoichiometry of binding. The 'N.D.' denotes that there is no detectable binding.

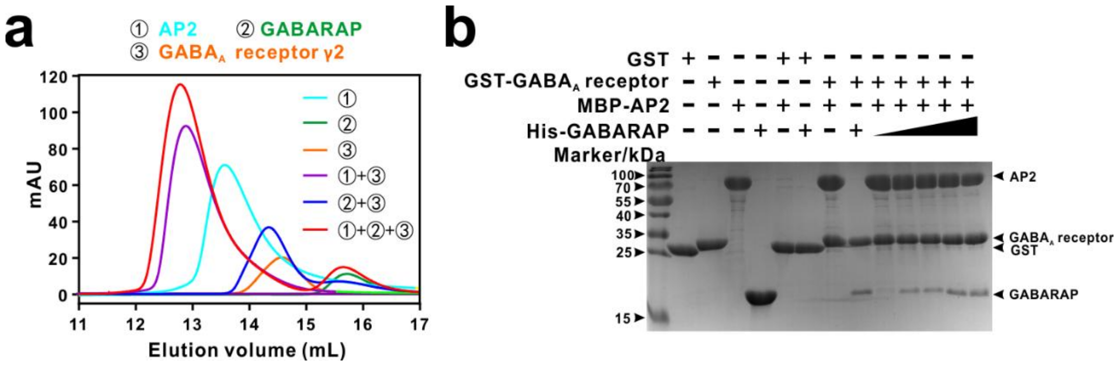

Supplementary Fig. 4 Competitive binding of GABARAP and AP2 to γ2-GIM.

(a) Analytic gel filtration and **(b)** competitive GST pull down assay of AP2, γ2-GIM, and GABARAP showing that GABARAP cannot compete with equimolar AP2 in binding with $GABA_A$ receptor, the experiments were repeated three times.

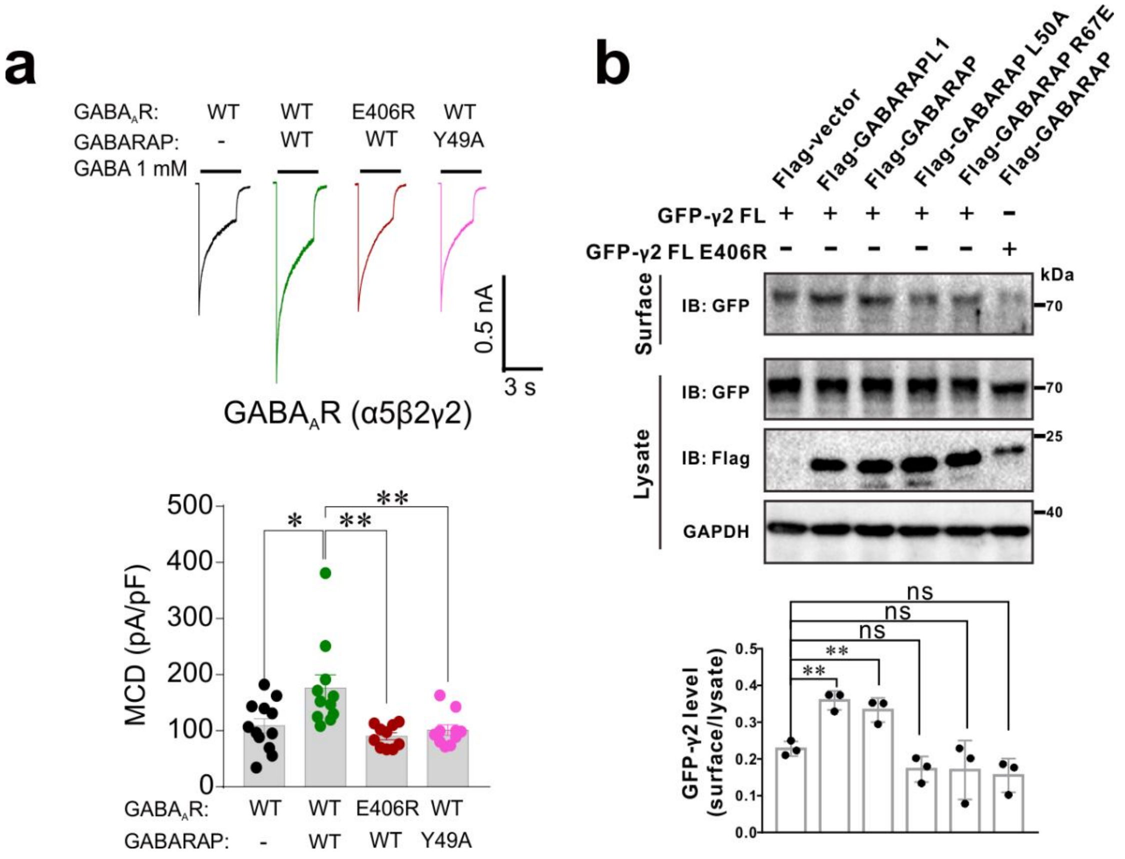

Supplementary Fig. 5 Specific interaction between GABARAP and GABA$_A$R is required for the GABARAP-mediated increase of GABA$_A$R membrane surface localization.

(a) Effects of GABARAP on GABA$_A$R-mediated current densities in HEK-293 cells co-expressing GABARAP and α5-containing GABA$_A$Rs. Representative trace records and average values of maximum current density activated by 1 mM GABA in HEK-293 cells co-expressing GABA$_A$Rs (α5β2γ2) and WT GABARAP or GABARAP mutants. n= n=12, 11, 10 and 10 of each group (from left to right). Data are represented as the mean ± SEM. * *P*= 0.0114, GABA$_A$R vs GABA$_A$R + GABARAP; ** *P*= 0.0016, GABA$_A$R + GABARAP vs GABA$_A$R^{E406R} + GABARAP; ** *P*= 0.0066, GABA$_A$R + GABARAP vs GABA$_A$R + GABARAP^{Y49A} based on One-Way ANOVA. **(b)** Surface biotinylation analysis of the GABA$_A$Rs. HEK-293 cells co-expressing GABA$_A$R (α1β2γ2) or GABA$_A$R (α1β2γ2^{E406R}) and WT GABARAP or GABARAPL1 or mutant GABARAP (L50A and R67E were selected) followed by cell surface biotinylation/immunoblotting. Data are represented as the mean ± SEM (n=3). ** *P*= 0.0022, GABA$_A$R + vector vs GABA$_A$R + GABARAPL1; ** *P*= 0.0091, GABA$_A$R +

vector vs $GABA_AR$ + GABARAP based on two tailed unpaired Student's *t* test; ns, not significant ($P > 0.05$).

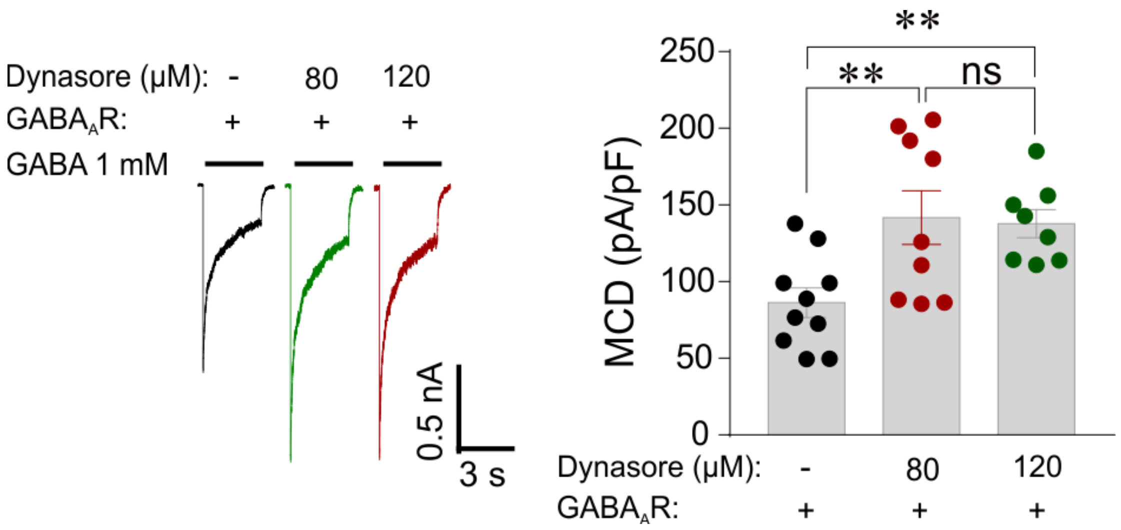

Supplementary Fig. 6 Effects of multiple dynasore concentrations on GABA$_A$R-mediated current densities in HEK-293 cells.

Representative trace records and average values of GABA currents activated by 1 mM GABA in HEK-293 cells expressing GABA$_A$R (α1β2γ2) with or without treatment using the endocytosis inhibitor dynasore (80 µM or 120 µM, 2h). n=10, 9 and 8 of each group (from left to right). Data are represented as the mean ± SEM. ** *P*= 0.0079, GABA$_A$R vs GABA$_A$R + 80 µM dynasore; ** *P*= 0.0076, GABA$_A$R vs GABA$_A$R + 120 µM dynasore based on One-Way ANOVA; ns, not significant (P > 0.05).

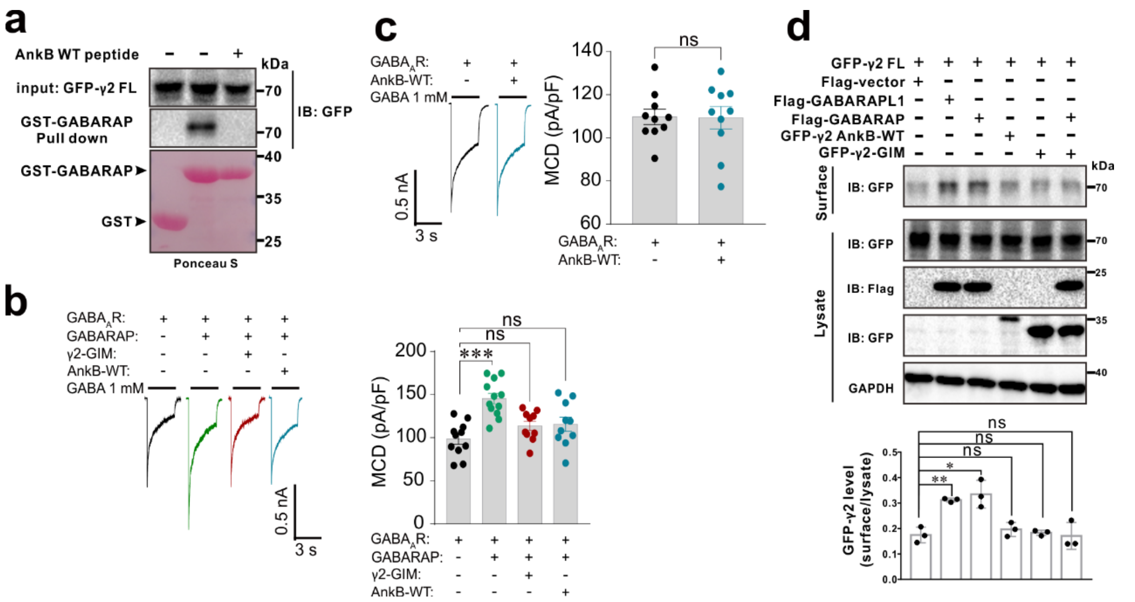

Supplementary Fig. 7 The AnkB WT peptide disrupts the GABARAP/γ2-GABA$_A$R interaction and blocks the GABARAP-mediated increase of GABA$_A$R membrane surface localization.

(a) GST pull-down assay showing that the AnkB WT peptide can effectively disrupt the GABARAP/γ2-GABA$_A$R interaction. **(b)** Representative trace records and average values of GABA currents activated by 1 mM GABA in HEK-293 cells co-expressing GABA$_A$R (α1β2γ2), GABARAP and γ2-GIM or AnkB-WT. n=11, 12, 10 and 10 of each group (from left to right). Data are represented as the mean ± SEM. *** $P = 0.0001$, GBA$_A$R vs GABA$_A$R + GABARAP based on One-Way ANOVA; ns, not significant (P > 0.05). **(c)** Representative trace records and average values of GABA currents activated by 1 mM GABA in HEK-293 cells co-expressing GABA$_A$R (α1β2γ2) and AnkB-WT. n=10. Data are represented as the mean ± SEM. ns, not significant (P > 0.05). **(d)** Surface biotinylation analysis of the GABA$_A$Rs. HEK-293 cells co-expressing GABA$_A$R (α1β2γ2) and GABARAP or GABARAPL1, AnkB WT peptide, γ2 398-415 peptide followed by cell surface biotinylation/immunoblotting. Data are represented as the mean ± SEM (n=3). ** $P = 0.0017$, GABA$_A$R + vector vs GABA$_A$R + GABARAPL1; * $P = 0.0114$, GABA$_A$R + vector vs GABA$_A$R + GABARAP based on two tailed unpaired Student's t test; ns, not significant ($P > 0.05$).

Supplementary Table 1. Statistics of X-ray Crystallographic Data Collection and Model refinement

**Data collection**	
Data sets	GABARAPL1/GABA$_A$R γ2-GIM
Space group	$P6_122$
Wavelength (Å)	0.9787
Unit Cell Parameters (Å)	a=b=89.38, c=115.00<br>α=β=90°, γ=120°
Resolution range (Å)	50-1.95 (1.98-1.95)
No. of unique reflections	19730 (967)
Redundancy	14.3 (13.9)
I/σ	14.4 (2.5)
Completeness (%)	96.4 (98.3)
R_{merge} [a] (%)	22.0 (96.1)
$CC_{1/2}$ (last resolution shell) [b]	0.775
**Structure refinement**	
Resolution (Å)	50-1.95 (2.05-1.95)
R_{cryst} [c]/R_{free} [d] (%)	19.74/23.15 (21.62/25.92)
rmsd bonds (Å) / angles (°)	0.008 / 0.967
Average B factor (Å^2) [e]	19.4
No. of atoms	
Protein atoms	2103
Water	84
Ligands	30
No. of reflections	
Working set	18748 (2642)
Test set	982 (158)
Ramachandran plot regions [d]	
Favored (%)	98.8
Allowed (%)	1.2
Outliers (%)	0

Numbers in parentheses represent the value for the highest resolution shell.

a. $R_{merge} = \Sigma |I_i - \langle I \rangle| / \Sigma I_i$, where I_i is the intensity of measured reflection and $\langle I \rangle$ is the mean intensity of all symmetry-related reflections.

b. $CC_{1/2}$ were defined by Karplus and Diederichs[1].

c. $R_{cryst}=\Sigma||F_{calc}| - |F_{obs}||/\Sigma F_{obs}$, where F_{obs} and F_{calc} are observed and calculated structure factors.

d. $R_{free}= \Sigma_T||F_{calc}| - |F_{obs}||/\Sigma F_{obs}$, where T is a test data set of about 5% of the total unique reflections randomly chosen and set aside prior to refinement.

e. B factors and Ramachandran plot statistics are calculated using MOLPROBITY[2].

Supplementary Table 2. The measured binding affinities between GABA$_A$R γ2-GIM and GABARAP/AP2 and their mutants based on ITC.

GABA$_A$ receptor γ2-GIM	GABARAP	K_d (μM)	AP2	K_d (μM)
WT	WT	4.9 ± 0.5	WT	0.25 ± 0.02
E402T	WT	8.4 ± 0.9	WT	0.56 ± 0.02
Y403E	WT	1.0 ± 0.1	WT	2.46 ± 0.22
Y405E	WT	N.D.	WT	ND
E406R	WT	N.D.	WT	0.69 ± 0.06
L408Q	WT	N.D.	WT	>50

Supplementary Table 3. List of primer sequences used in this study.

Primer name	Primer sequences (5'-3')
γ2-1-BamH1-up	gcggatccatgagttcgccaaataca
γ2-466-EcoR1-dn	cggaattcttacagataaagataggagac
γ2-375-BamH1-up	gcggatcccctgcccctaccattgat
γ2-397-Xhol1-dn	cgctcgagttattgaaggtgtgtggcatt
γ2-398-BamH1-up	gcggatccgagagggatgaagaatat
γ2-415-Xhol1-dn	cgctcgagttaactggcacagtccttgcc
γ2-404-BamH1-up	gcggatccggctatgagtgtttggat
γ2-424-Xhol1-dn	cgctcgagttatcggcaatcttcaaaaca
γ2-424-BamH1-up	gcggatcccgaacaggagcctggaga
γ2-441-Xhol1-dn	gcctcgagttagtccattttggcaatgcg
γ2-E402T-up	caagagagagatgaaacatacggctatgagtgtctggacg
γ2-E402T-dn	cgtccagacactcatagccgtatgtttcatctctctcttg
γ2-Y403E-up	caagagagagatgaagaggagggctatgagtgtctggacg
γ2-Y403E-dn	cgtccagacactcatagccctcctcttcatctctctcttg
γ2-Y405A-up	gagagagatgaagagtacggcgccgagtgtctggacggcaaggac
γ2-Y405A-dn	gtccttgccgtccagacactcggcgccgtactcttcatctctctc
γ2-E406R-up	agagatgaagagtacggctatcgctgtctggacggcaaggac
γ2-E406R-dn	gtccttgccgtccagacagcgatagccgtactcttcatctctctc
γ2-L408Q-up	gatgaagagtacggctatgagtgtcaagacggcaaggac
γ2-L408Q-dn	gtccttgccgtcttgacactcatagccgtactcttcatc
GABARAP-1-BamH1-up	gcggatccatgaagttcgtgtacaaa
GABARAP-117-EcoR1-dn	cggaattctcacagaccatagacgctttc
GABARAP-E17A-up	gaagcgccgctctgccggcgagaaaatccg
GABARAP-E17A-dn	cggattttctcgccggcagagcggcgcttc
GABARAP-K24E-up	gcgagaaaatccgaaaggagtacccagaccgggtccc
GABARAP-K24E-dn	gggacccggtctgggtactcctttcggattttctcgc
GABARAP-Y49A-up	gacaaaaagaaagccctggtgccttctgat
GABARAP-Y49A-dn	atcagaaggcaccagggctttctttttgtc
GABARAP-L50A-up	gacaaaaagaaatacgccgtgccttctgat
GABARAP-L50A-dn	atcagaaggcacggcgtatttctttttgtc
GABARAP-K66E-up	tctacttcttgatccgggagcgaattcatctccgt
GABARAP-K66E-dn	acggagatgaattcgctcccggatcaagaagtaga
GABARAP-R67E-up	attctacttcttgatccggaaggagattcatctccgtgctgaagatg
GABARAP-R67E-dn	catcttcagcacggagatgaatctccttccggatcaagaagtagaat
GABARAPL1-1-BamH1-up	gcggatccatgaagttccagtctaag
GABARAPL1-117-EcoR1-dn	cggaattctcattttccatagacactttc
GABARAPL1-K24E-up	gaaggtgaaaagattaggaaggagtacccggaccgggtgc
GABARAPL1-K24E-dn	gcacccggtccgggtactccttcctaatcttttcaccttc
GABARAPL1-L50A-up	ggataagaggaagtacgctgtgccctccgacctc
GABARAPL1-L50A-dn	gaggtcggagggcacagcgtacttcctcttatcc

GABARAPL1-R67E-up	ctacttcttaatccggaaggagatccacctgagacctgag
GABARAPL1-R67E-dn	ctcaggtctcaggtggatctccttccggattaagaagtag
GABARAPL2-1-BamH1-up	gcggatccatgaagtggatgtttaag
GABARAPL2-117-EcoR1-dn	cggaattcttagaagccaaaagtgttctc
LC3A-1-BamH1-up	gcggatccatgccctccgaccggcct
LC3A-121-Xhol1-dn	cgctcgagttagaagccgaaggtttcttg
LC3B-1-BamH1-up	gcggatccatgccgtccgagaagacc
LC3B-125-Xhol1-dn	cgctcgagttacacagccattgctgtccc
LC3C-1-BamH1-up	gcggatccatgccgcctcccacagaaa
LC3C-147-Xhol1-dn	cgctcgagttagagaggattgcagggtct
AP2 μ1-158-BamH1-up	gcggatcccaaattggctggaggcggg
AP2 μ1-435-EcoR1-dn	cggaattcttagcagcgggtttcgtaaat
α1-1-BamH1-up	gcggatccatgaagaaaagtcggggtct
α1-455-EcoR1-dn	cggaattcttattgatggggtgtgggggc
β2-1-BamH1-up	gcggatccatgtggagagtgcggaaaag
β2-512-EcoR1-dn	cggaattcttagttcacataataaagcca

REFERENCES

1. Karplus PA, Diederichs K. Linking crystallographic model and data quality. *Science*. **336**, 1030-1033 (2012).
2. Chen, V.B. et al. MolProbity: all-atom structure validation for macromolecular crystallography. *Acta Crystallogr D Biol Crystallogr* **66**, 12-21 (2010).
